# Conditions Successfully Treated by Electrolysis

**Published:** 1899-12

**Authors:** M. B. Hutchins

**Affiliations:** Atlanta, Ga.


### CONDITIONS SUCCESSFULLY TREATED BY ELECTROLYSIS.*

BY M. B. HUTCHINS, M.D.,

ATLANTA, GA.

It is proper that this short paper begin with a brief explanation of the technique of electrolytic work.

* Read before the Atlanta Society of Medicine, October, 1899.

A galvanic battery is used, not a faradic, as I know one physician tried. Neither can hairs be destroyed properly with the galvano-cautery point, as one of my out-of-town correspondents thought.

The negative pole is used for the attachment of the needle. The action of the current through the needle attached to the negative pole is very like that of an alkaline caustic, but a caustic action which is almost absolutely controllable and definable. The action of the positive pole is like that of an acid caustic. Negative electrolysis is solvent, positive is coagulant.

A milliammeter is absolutely necessary for accurate and safe work. The resistance of the hands and tissues of patients is extremely variable and cannot be known without a meter. It is also desirable to have a rheostat in circuit, to obtain finer graduation of current than is possible with a simple switch-board, to increase or decrease the current.

The action of the current is antiseptic, and it is only necessary to keep the needles clean, and prevent secondary infection by means of proper local treatment.

Destruction of various abnormal growths by electrolysis is very tedious, but patients prefer it to the more rapid methods of knife and caustic, and it is the *only* safe means of removing superfluous hairs. Moles and other smaller growths are removed by this method with less danger of infection and less scar than by any other method.

The pain experienced by patients is very variable. Many do not complain at all, others only at the beginning; the electricity in most cases producing anesthesia, which sometimes appears to last for many days in the parts treated. A few patients complain of the pain and never get accustomed to it. Some say that a weak current gives more pain than a strong, the only explanation being that the stronger affords more anesthesia. After-treatment of the electrolysed parts is by means of watery, cooling, antiseptic lotions, or, if there is crusting, a greasy application.

After trying the various needles made for the work, I have returned to the small sewing needle for most cases, while for the destruction of larger growths or angiomata I use a bayonet-pointed, gold-plated needle.

If a purely coagulant effect is desired the positive pole is used, in all other cases the negative. A current strength of $\frac{1}{4}$ to 1 milliampere is used, according to the resistance of the tissues treated. If a weak current is employed the application is more prolonged, but too long passage of a weak current through a point will give much the same effect as a briefer application of a strong current.

For the destruction of moles and similar small growths, the current is applied through a needle inserted in the growth in such direction as the shape of the lesion requires. The punctures should not be so close together as to permit confluence of the minute eschars or crusts. It is best to take several weeks for the treatment if the best result as to appearance is desired. Warts require deep insertion of the needle and a strong current, as they have a way of regrowing if the slightest particle is left. Sometimes the pit left after their total destruction by electrolysis of the base will fill up.

Small angiomata can be destroyed by negative electrolysis through the feeding vessels. I have been disappointed in the results of efforts to coagulate the blood in these tumors by means of positive electricity. Small dilated vessels are destroyed by perpendicular or longitudinal puncture and a rather weak negative current.

Much has been written about the electrolytic destruction of superfluous hairs. Every man seems to have his own method, but the principle is the same. The negative current must be applied to the root and papilla of the hair long enough to permanently destroy their function. The location of the root is a matter of judgment, almost of guess, and I do not believe any operator can ever become so skilled as to be able to do this work perfectly. To the trained touch the contact of the needle with the bottom of the follicle is easily perceptible, but if the hair bends after entering the skin, as the majority do, the needle point may go into the sebaceous gland, or against the curved side of the follicle. Scarring will follow the treatment if the current action is sufficient to necrose much of the true skin. Vesication around the needle will usually give warning of too intense action of the current. Proper local treatment will prevent inflammation or scarring in many cases in which these would occur if not treated. At best, it is mean and trying work, and poorly paid, and deserves to exist only because it is the only way to get rid of the disfiguring hairs.

The introduction to this paper having been longer than I intended I shall be more brief in the part which corresponds to its title.

A congenital mole on the forehead of a young girl was about $\frac{3}{4}$ x $\frac{3}{4}$ inch in diameter and its highest part about $\frac{1}{6}$ of an inch. The lesion was brown and there were many fine hairs growing in its surface. This growth was destroyed, after many treatments, with such success that its site was but slightly perceptible.

Lesions known as "benign cystic epitheliomata" destroyed by deep electrolysis through the base.

A perfect result in a fibrous scar on the carmine border of the lower lip, which had been somewhat reduced by actual cautery, but was still annoying to the patient.

A congenital, soft, linear nevus along the right jaw border on a lady's face.

A false aneurysm in the border of the palm of a young woman's hand, which had its site in an injured fat lobule; destroyed by negative electrolysis of its blood-vessels, after failure of ligation and excision.

Small, cavernous angiomata of the skin, successfully treated by the same method.

Dilated vessels on the face destroyed, but often all collateral radicles had to be included to prevent establishment of new circulation with dilatation.

The chamois-skin-colored lesions on the eyelids, known as xanhomata, I have also removed satisfactorily.

A triangular patch of pale, freckle-like pigmentation, with a base of finely dilated vessels, occupying the right lower jaw border and sub-maxillary regions, probably for the most part congenital, was greatly improved by electrolysis. This was the only method of treatment which promised anything and the result obtained showed that persistence in the treatment would have produced a satisfactory result.

I have lately removed, without perceptible scar, a very superficial epithelioma of the right lower eyelid and conjunctival border, the situation of the lesion justifying the trial of this rather than harsher measures.

By producing a small electrolysis eschar, including powder grains imbedded in the skin, I have been able to remove them when picking and blistering failed, but the treatment is rather unsatisfactory.

Numerous cases of "superfluous hairs," involving various regions have been treated with more or less success. Some cases gave but little further trouble, others had a certain number of returns which, added to the reserve supply of fine ones destined to grow coarse, rendered necessary repeated operations. A writer recently stated that he had removed two mustaches and one full beard from ladies with no returns. I doubt his statement.

Many moles have been removed, leaving in most cases practically no trace of their former presence. Treating moles by electrolysis does not cause them to become cancerous. Leaving them affords a good site for cancer to begin. Level pigmentations are satisfactorily removed by careful electrolysis.

Soft warts, such as the sebaceous variety and the so-called venereal, yield very rapidly to this treatment. The hard and horny variety must be treated with a strong enough current to obtain the effect of caustic potash, and to such depth that not a particle will remain to furnish the germ of new growth. One wart treated as above sloughed out leaving a pit which, however, very soon filled up.

A small chondroma of the edge of the left auricle was satisfactorily treated by means of negative electrolysis.

I have given you, from memory, a list of over fifteen conditions successfully treated by electrolysis, most of them during the past ten or twelve months. A reference to my books would probably discover a few more cases of lesions other than mentioned.

The work requires great caution for its successful performance, is exceedingly tedious and trying to operator and patient, and slow in the attainment of final results, but it is the only satisfactory treatment for many conditions and offers the best chance of a pleasing cosmetic result.

*Nos. 305–306 Fitten Building.*

